# Photo fermentative biohydrogen production potential using microalgae–activated sludge co-digestion in a sequential flow batch reactor (SFBR)†

**DOI:** 10.1039/d2ra06014k

**Published:** 2022-10-18

**Authors:** Muhammad Asad Javed, Ashraf Aly Hassan

**Affiliations:** Department of Civil and Environmental Engineering, United Arab Emirates University Al Ain 15551 United Arab Emirates alyhassan@uaeu.ac.ae; National Water and Energy Center, United Arab Emirates University Al Ain 15551 United Arab Emirates

## Abstract

Biohydrogen (bioH_2_) is a sustainable energy source that can produce carbon-free energy upon combustion. BioH_2_ can be generated from microalgae by photolytic and anaerobic digestion (AD) pathways. The AD pathway faces many challenges when scaling up using different bioreactors, particularly the continuous stirred tank reactor (CSTR) and sequential flow batch reactor (SFBR). Therefore, the performance characteristics of SFBR were analysed in this study using *Chlorella vulgaris* and domestic wastewater activated sludge (WWAS) co-culture. An organic loading rate (OLR) of 4.7 g COD L^−1^ day^−1^ was fed to the SFBR with a hydraulic retention time (HRT) of five days in the presence of light under anaerobic conditions. The pH of the medium was maintained at 6 using a pH controller for the incubation period of 15 days. The maximum bioH_2_ concentrations of 421.1 μmol L^−1^ and 56.6 μmol L^−1^ were observed in the exponential and steady-state phases, respectively. The effluent had an unusually high amount of acetate of 16.6 g L^−1^, which remained high with an average of 11.9 g L^−1^ during the steady state phase. The amount of bioH_2_ produced was found to be inadequate but consistent when operating the SFBR with a constant OLR. Because of the limitations in CSTR handling, operating a SFBR by optimizing OLR and HRT might be more feasible in operation for bioH_2_ yield in upscaling. A logistic function model was also found to be the best fit for the experimental data for the prediction of bioH_2_ generation using co-culture in the SFBR.

## Abstract 2

Biohydrogen (bioH_2_) is a sustainable energy source that can produce carbon-free energy upon combustion.

## Graphical abstract

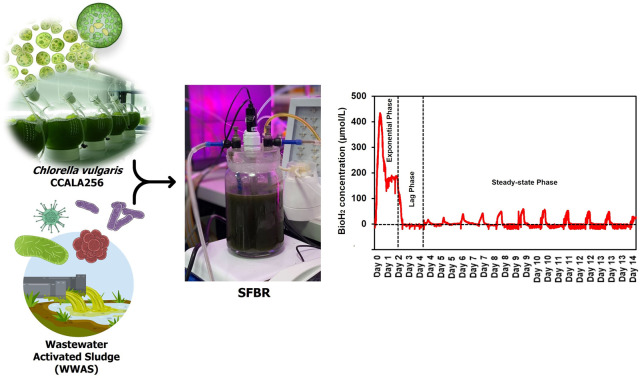

## 1. Introduction

The substitution of fossil fuels with other sources of energy has become a more and more critical issue in the world's energy revolution, with a major driving force changing the paradigm towards renewable energy.^1^ At present, a sustainable goal is low-cost biological hydrogen gas (bioH_2_) production using microalgae as a feedstock (dry biomass) or as photosynthetic microorganisms (living culture).^2,3^ Hydrogen (H_2_) has a high energy content and as a biofuel it does not emit carbon dioxide (CO_2_).^4^ On the other hand, biomethane contributes toward greenhouse gas (GHG) emissions.^5^ Efforts to utilize lignocellulosic feedstocks and photosynthetic microorganisms for biological conversion to bioH_2_ have focused primarily on anaerobic digestion (AD) and secondarily on biophotolysis.^6,7^ The AD of three microalgae genera, *Chlorella*, *Chlamydomonas*, and *Scenedesmus* sp., has been extensively researched due to their immense potential for producing bioH_2_ and biogas.^8–10^ Moreover, it has been reported that AD is a viable strategy for producing bioenergy from microalgae.^11^ However, there are several constraints such as environmental factors, microalgal species, input/output energy waste, oxygen (O_2_) regulation, nutrient level, external substrates, light intensity, and pH which might hamper the performance efficiency of microalgae.^6^ The co-culturing of microalgae and bacteria, in this case, is a promising technique to regulate O_2_ which is one of the constraints during microalgal bioH_2_ production.^12^ However, using waste resources such as activated sludge as a source of bacteria has also shown a significant enhancement in bioH_2_.^3,13^ Thus, *Chlorella vulgaris* has been observed to have a higher biodegradation ability than in the earlier studies with a high conversion efficiency of 50%.^14^ A low conversion efficiency of 30% has also been reported for digestion by *Scenedesmus obliquus*.^15^ A 9.4% biodegradation ability of non-pretreated *Scenedesmus* sp. was also seen under mesophilic conditions in a continuously stirred tank reactor (CSTR).^16^

The bioH_2_ production through batch reactors suffers from a variety of inhibitory elements during anaerobic digestion, such as the accumulation of volatile fatty acids (VFAs) in the medium, low pH at the end of incubation, degradation of microalgal cells, reduction in the amount of chlorophyll, and reduced consumption of carbon substrates.^3^ These factors reduce the efficiency of AD metabolism and result in the ceasing of bioH_2_ production after a few days. Compared to bioH_2_ generation through batch reactors, bioH_2_ yield through continuous or sequential reactors is much more effective in reducing the inhibitory factors and sustaining the bioH_2_ production for a more extended period, and is less laborious in terms of medium replacement.^17^ CSTRs are widely utilized for bioH_2_ generation; nevertheless, their performance is still restricted due to biomass loss and poor carbon substrate consumption.^18^ To avoid such metabolic inhibitory pathways during the batch and continuous fermentation processes, a sequential flow batch fermentation reactor was proposed for cultivation and continuous bioH_2_ generation using glucose/xylose.^19,20^ The inflow and outflow of fermentation broth occurs simultaneously during the fermentation process in continuous or sequential flow reactors. Consequently, the inhibitory factors such as substrate inhibition, nutrient deprivation, VFA dilution, and further metabolite repression can be effectively alleviated. Furthermore, a high biomass density can be maintained in the reactor, implying a strong potential for sustained bioH_2_ generation.

There are several types of reactors, such as the anaerobic membrane bioreactor (AnMBR), upflow anaerobic sludge blanket reactor (UASB), sequential flow batch reactor (SFBR), and CSTR, which are commonly used for microalgal/lignocellulosic and wastewater co-digestion operated under mesophilic and thermophilic conditions.^21–23^ It has been reported that using an AnMBR can lead to 35% enhanced methane yield and 69% biodegradability in microalgae co-digestion without ammonia inhibition.^24^ Similarly, another study has reported 65–73% biodegradability in microalgae–sludge co-digestion using an AnMBR in the mesophilic temperature range.^23^ It has further been observed that continuous bioH_2_ production performance depends more on pH than hydraulic retention time (HRT) by co-digesting lignocellulosic substrates with liquid manure in an anaerobic sequencing batch reactor.^25^ The UASB reactor has been reported for effective and cost-efficient wastewater treatment in co-digestion with microalgae biomass, resulting in organic matter removal of 61–63% chemical oxygen demand (COD) and 74% total suspended solids (TSS).^26^

There is a limited number of previous studies which investigated the co-culture of microalgae and mono bacterial strains for oxygen scavenging in batch reactors.^10,27,28^ However, in the present work, real domestic wastewater activated sludge (WWAS) was adopted as a bacterial partner for co-digestion with *Chlorella vulgaris* to study the semi-continuous bioH_2_ generation using a sequential flow batch reactor (SFBR). The bacteria present in the activated sludge served the purpose of taking up O_2_ and maintaining the anaerobic environment. The lab scale SFBR was operated with microalgae and WWAS photo fermentative co-culture under mesophilic conditions to analyse the effects of a constant organic loading rate (OLR), hydraulic retention time (HRT), total organic carbon (TOC), and VFAs, mainly acetate, on bioH_2_ production. Some of the previous experimental studies used an anaerobic sequencing batch reactor (ASBR) for anaerobic hydrogen production from different types of wastewater and food waste.^29–33^ However, to the authors' knowledge, the current study unconventionally employed the SFBR to determine the bioH_2_ potential of microalgae and WWAS photo fermentative co-culture based on glucose as a carbon substrate.

## 2. Materials and methods

### 2.1. Co-culture of *Chlorella vulgaris* and WWAS

The *Chlorella vulgaris* CCALA 256 (*C. vulgaris*) strain was obtained in agar media from a culture collection laboratory (CCALA) in the Czech Republic (https://ccala.butbn.cas.cz/). The purity of the strain was checked microscopically twice a week during the culturing and the strain was found to be uncontaminated at the time of harvesting. The culture was cultivated at pH 7 in the specified Z medium, which included all the extra nutrients required for microalgae growth, as reported in Table S1 (ESI).† The stock culture was grown in autoclaved 5 L Schott bottles with constant aeration and light with a 12 : 12 h (light : dark) cycle. Continuous stirring was carried out at 200 rpm and light of *λ* 450 and 650 nm (red and blue) was provided at room temperature for homogenous light provision and air mixing in culture. The growth conditions were maintained until the wet algal biomass reached 13.2 mg mL^−1^.

The domestic WWAS was obtained from the Al-Saad wastewater treatment plant in Al Ain, United Arab Emirates. The wet organic biomass concentration in activated sludge was 98.3 mg mL^−1^. The activated sludge was kept in a non-transparent container in a refrigerator to prevent microbial growth from altering its composition before usage. The shelf life of the WWAS was limited to one month to prevent microbial changes. Table 1 shows the initial characteristics of the *C. vulgaris* CCALA 256 culture and WWAS utilized in this investigation.

**Table 1:** Initial characteristics of microalgae (*C. vulgaris* CCALA 256) and domestic wastewater activated sludge (WWAS)

Parameter	Microalgae	Activated sludge
pH	8.1	7.6
TS (g L^−1^)	12.1	18.0
VS (g L^−1^)	11.7	15.1
COD (g L^−1^)	4.5	17.6
Cl^−^ (mg L^−1^)	307.0	86.9
PO_4_^3−^ (mg L^−1^)	173.6	17.5
SO_4_^2−^ (mg L^−1^)	135.4	—
NO_3_^−^ (mg L^−1^)	—	526.1
Na (%-V)	80.5	71.8
Mg (%-V)	19.2	27.8
Ca (%-V)	0.2	0.4
Other trace elements (K, Mn, Fe, Co, Ni, Cu, Zn) (%-V)	<1	<1

The bioH_2_ generation was evaluated using an inoculum ratio of 1 : 1.5 v/v (microalgae : WWAS) with 23.5 g COD L^−1^ at the start of the experiment. Glucose was supplied at 10 g L^−1^ as an exogenous carbon substrate. The harvested microalgae and WWAS were centrifuged at 8000 rpm for 10 minutes before co-culturing. The supernatant was discarded, and the wet biomass was collected and washed with deionized (DI) water before resuspension in TAP medium, as shown in the recipe provided in Table S2 (ESI)†.

### 2.2. SFBR setup and operation

**Fig. 1:**
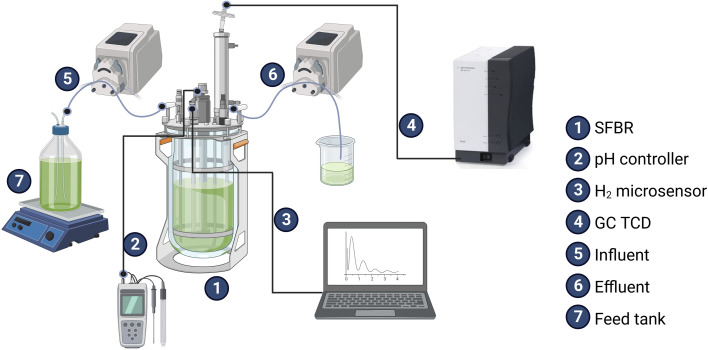
A schematic of the studied SFBR unit setup.

A transparent lab-scale SFBR of 500 mL working volume and 100 mL headspace volume was constructed to operate in sequential bioH_2_ generation mode, as shown in Fig. S1 (ESI†). The reactor was filled with a 500 mL medium of inoculum ratio 1 : 1.5 v/v purged with high-quality 99.9% nitrogen (N_2_) gas for 10 minutes to create the anaerobic conditions before sealing the reactor. Five sampling ports were installed in the SFBR to monitor the pH, continuous bioH_2_ concentration, inflow, and outflow of the medium, as shown in Fig. 1.

First, the SFBR was operated in batch mode for 24 h to completely turn the system into an anaerobic digestion phase and accumulate biomass; it was then changed to sequential batch mode for the rest of the incubation period. The SFBR operation was divided into three phases: I-exponential phase, II-lag phase, and III-steady state phase. The SFBR was fed every day with an OLR of 4.7 g COD L^−1^ (100 mL of inoculum). The hydraulic retention time (HRT) of the reactor was selected to last five days. Continuous light of illumination intensity 6921 μmol m^−2^ s^−1^ was provided around the SFBR in order to avoid the shading effect. The SFBR was placed on a magnetic stirrer plate at 400 rpm for homogeneous mixing of the inoculum and of the influent with the inoculum. The temperature of the reactor was maintained in the mesophilic range under 36 °C during the whole incubation period.^34^ The pH was automatically maintained at 6 by a pH controller using a 1 M NaHCO_3_ buffer solution.^35^

### 2.3. Analytical method

The bioH_2_ concentration (μmol L^−1^) profiling was carried out using an H_2_S insensitive H_2_ microsensor (H_2_-X-50, Unisense A/S, Århus, Denmark). The gas composition was determined by gas chromatography using a thermal conductivity detector (TCD) based 490 Micro gas chromatograph (GC) (Agilent Technologies Inc., CA, USA) with argon as the carrier gas operated with 20 m Molsieve 5 A and 10 m PoraPLOT Q columns. The method developed to run the Micro GC was set to take injections at column and injector temperatures of 80 °C and 50 °C, respectively. A TOC analyzer (Analytik Jena multi N/C 2100) was used to determine the TOC concentration (g L^−1^). The acetate concentration (g L^−1^) was determined by ion chromatography using a Thermo Scientific Dionex Aquion AS-DV IC equipped with a Dionex IonPac AS22 (4 × 250 mm) analytical column and a Dionex IonPac AG22 (4 × 50 mm) guard column. The pH was maintained and controlled by using a Bluelab pH Controller Connect – CONTPHCON (Bluelab, Tauranga, New Zealand) with a probe continuously dipped inside the medium in the SFBR.

### 2.4. Monitoring and data collection

The TOC and acetate concentrations were measured daily by taking samples from the SFBR effluent. The concentration of bioH_2_ in the generated gas during the photofermentation (PF) was monitored through a H_2_ microsensor. The bioH_2_ concentration data were continually retrieved every second. However, to check the volume of the gas generated, the gas was directed to a respirometer equipped with specially designed cells that registers the total volume of gas produced. Afterward, the gas passed through a 16-port actuator valve to avoid mixing with external ambient air prior to the gas chromatography. The pH was continuously monitored and controlled using a pH controller *via* a built-in peristaltic pump that precisely controls and maintains the pH at a set level by adding alkali or acid.

## 3. Results and discussion

### 3.1. Biogas production potential using the SFBR

**Fig. 2:**
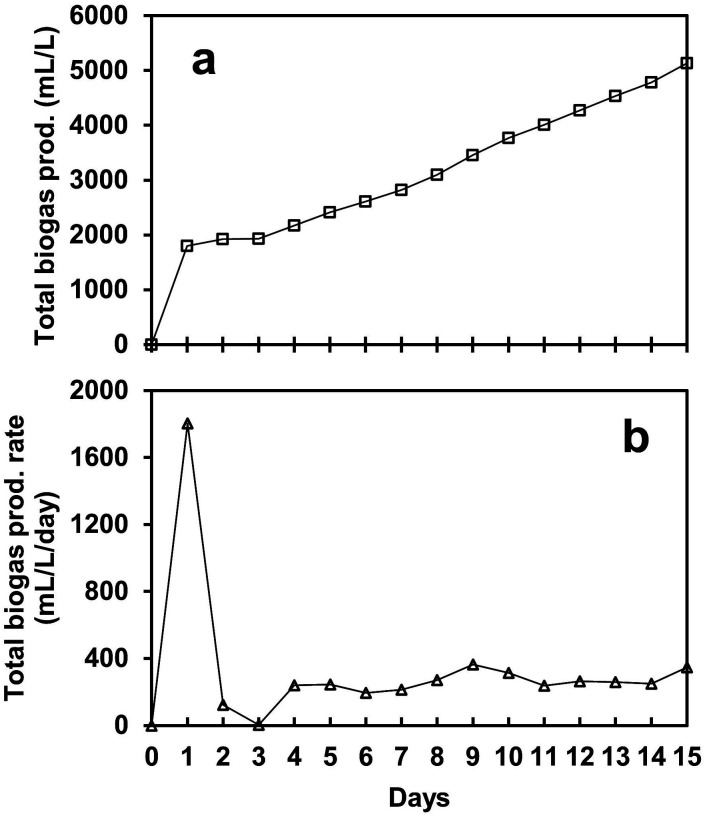
The profile of biogas production (a) and biogas production rate (b) during the incubation period of the SFBR operation.

Fig. 2a shows the biogas production achieved by the SFBR using a co-culture mixture of microalgae and WWAS. The gas production was influenced by the OLR containing glucose as a carbon substrate. After inoculating the SFBR with co-culture, the bioreactor was operated in batch mode for 24 h, and subsequently with the sequential addition of 4.7 g COD L^−1^ day^−1^ for an incubation period of 15 days. It was observed that 5134 mL L^−1^ of total biogas was generated in 15 days of photo fermentation, as shown in Fig. 2a. After a lag phase of two days, the SFBR achieved steady-state conditions and started producing an average of 267 mL L^−1^ day^−1^ of biogas of biogas, as shown in Fig. 2b.

As a constant OLR was used during the whole incubation period, the total biogas production rate remained relatively stable between 194 mL L^−1^ day^−1^ and 364 mL L^−1^ day^−1^ during the steady-state phase. The maximum biogas production rate of 364 mL L^−1^ day^−1^ was observed on day 9, and the minimum of 194 mL L^−1^ day^−1^ was observed on day 6 during the steady-state phase. It was observed that during the exponential phase, the SFBR produced the highest yield of 1803 mL L^−1^ day^−1^ of biogas, followed by a lag phase during which the gas production reduced to almost zero. However, afterward, the SFBR started producing gas in a pattern until the process stopped on day 15.

The results suggest that the microbial community acclimatized to the anaerobic environment in the first two phases and continued to produce biogas in a pattern afterward. The variation in biogas production after the lag phase shows that the co-culture easily degrades the substrate depending on the organic carbon available and consumed during AD. The sequential gas production, however, indicates that as the inoculum is supplied to the reactor, the concentration of degradable organic matter rises, resulting in biogas production unless the process inhibitors dominate the metabolic process. The main metabolic process parameters such as pH, VFAs (acetate), and TOC should be maintained in a favourable range to avoid system overload. This also suggests that the co-culture of microalgae and WWAS in the SFBR is a sustainable sequential bioH_2_ production strategy. However, the operational control parameters such as C/N, OLR, and HRT, along with VFA removal, must be adjusted at an appropriate level for maximum utilization of co-culture bioH_2_ potential and substrate degradation in the SFBR.^29,30,36^

### 3.2. Viability of sequential bioH_2_ generation in an SFBR

**Fig. 3:**
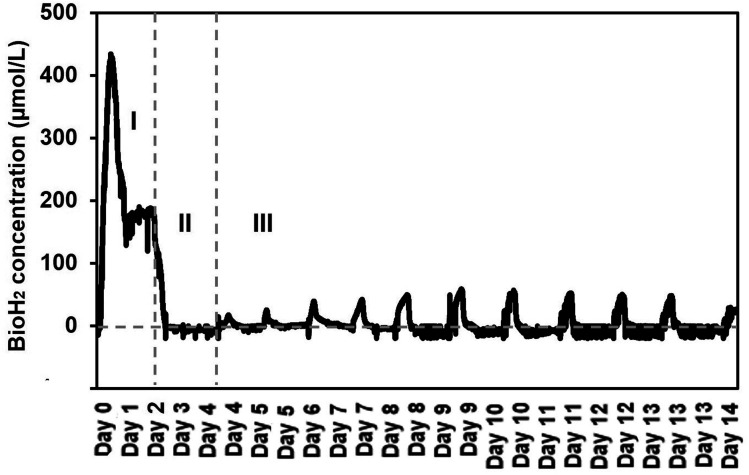
The sequential bioH_2_ production in the SFBR during phase I (exponential), II (lag), and III (steady-state).

It was found that the SFBR was consistent in bioH_2_ production with a constant OLR of 4.7 g COD L^−1^ day^−1^. The bioH_2_ concentration reached the highest value of 421.1 μmol L^−1^ in the exponential phase (phase I) of the first 24 h when the SFBR was operating in batch mode, as shown in Fig. 3. Following that, a lag phase (phase II) was observed for two days, during which the bioH_2_ concentration dropped to zero while the 100 mL of inoculum in the SFBR was replaced with fresh inoculum, as also illustrated in Fig. 3. The steady-state phase (phase III) was the sequential bioH_2_ production phase during which peaks of 56.4 and 54.4 μmol H_2_ L^−1^ were observed on days 9 and 10, respectively, as shown in Fig. 3. Fig. 3 shows that during the steady-state phase, there was a steady increase in bioH_2_ concentration from 15.1 μmol L^−1^ on day 4 to a maximum of 56.4 μmol L^−1^ on days 9 and 10, followed by a gradual decline to 28 μmol L^−1^ at the end of 15 days of incubation.

It is also evident from the results that the SFBR performed poorly compared to the results in the exponential phase during which the SFBR was operated in batch mode and performed significantly. This is likely due to the lower concentration of VFAs during the exponential phase, whereas the high yield of VFAs, mainly acetate, during the lag and steady-state phases may oversaturate the medium and hinder the bioH_2_ yield. Although the pH of the medium was maintained at 6 continuously, due to the high concentration of acetate in the medium inside the SFBR, there was a relatively reduced yield of sequential bioH_2_ production as compared to the yields reported in some of the previous studies.^29,30^

### 3.3. Biogas composition during SFBR operation

The concentrations of major biogas components, namely H_2_, O_2_, N_2_, CH_4_, and CO_2_, from microalgae and WWAS co-culture, are given in Table 2. The bioH_2_ content was significantly higher in the exponential phase, up to 9.4%, compared to the lag and steady-state phases. The N_2_ content in the main biogas stream was the highest (48–68%), followed by O_2_ and CO_2_ with concentrations of 12–20% and 3.3–10%, respectively, during the whole incubation period. The low concentration of bioH_2_ and CO_2_ in the SFBR compared to the batch mode is due to the high concentration of O_2_ in the biogas stream, which is expected to be produced by the photosynthetic metabolism of microalgae in the presence of light. This shows that the environment within the SFBR was partly aerobic during the whole incubation period, and favourable conditions for AD did not develop, resulting in lower bioH_2_ generation. The highest concentration of CO_2_ in the exponential phase also shows that the fermentation process occurred, and the highest concentration of bioH_2_ was registered. However, the low CO_2_ content during phases II and III, on the other hand, indicates diminished fermentative metabolism. The fraction of CH_4_ (<1%) was also detected (Table 2), and was responsible for the methanogenic activity.

**Table 2:** The concentration (%) of different gases in the biogas stream obtained by photo fermentation of algae and activated sludge co-culture using an SFBR

Days	H_2_ (%)	O_2_ (%)	N_2_ (%)	CH_4_ (%)	CO_2_ (%)
0	—	—	—	—	—
1	9.41	11.97	47.76	—	9.58
2	—	—	—	—	—
3	—	—	—	—	—
4	0.02	19.73	73.85	—	3.91
5	0.04	16.83	63.28	—	3.96
6	0.01	18.97	70.93	—	3.37
7	—	20.36	75.95	—	0.05
8	0.32	15.57	59.33	—	7.71
9	0.33	16.15	61.39	0.05	7.74
10	0.63	16.11	61.08	0.07	4.75
11	0.51	17.89	67.94	0.05	0.82
12	0.75	16.36	63.16	0.09	4.07
13	0.81	16.46	60.28	0.06	3.54
14	0.51	17.77	64.17	0.04	4.12
15	0.67	16.93	62.71	0.03	5.03

**Fig. 4:**
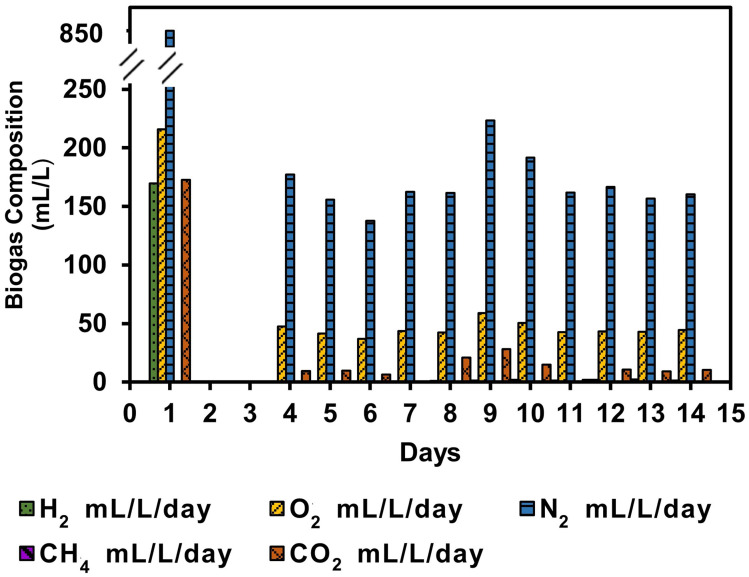
The variation in biogas composition on a daily basis in the SFBR.

The bioH_2_ was observed to be evident on day 1 in a concentration of 170 mL L^−1^ with almost the same volume of CO_2_ of 172 mL L^−1^, as shown in Fig. 4. The lag phase of days 2 and 3 shows an almost negligible volume of gas which was not analysed. However, the gas composition during the steady-state phase registered an average of 45 mL O_2_ L^−1^ day^−1^, 169 mL N_2_ L^−1^ day^−1^, and 11 mL CO_2_ L^−1^ day^−1^. The inoculum ratio of 1 : 1.5 v/v was optimised for maximum bioH_2_ generation in batch reactors;^3^ however, the same ratio did not retain or improve the bioH_2_ yield in the SFBR. It was also observed that with an HRT of five days, the bacterial partner in microalgae–WWAS co-culture did not work significantly in consuming the O_2_ produced by microalgae. An HRT of 12 h improved the bioH_2_ yield to a maximum of 0.51 mol H_2_ mol^−1^ glucose added in the ASBR process,^30^ therefore, the HRT should be optimized to improve the bioH_2_ concentration during SFBR operation.

### 3.4. Operational control parameters

The biological process performance of the SFBR was evaluated by monitoring three operational control parameters daily: pH, TOC, and acetate concentration. It was observed that when the SFBR was fed on a daily basis, the pH started dropping, which was likely due to the production of acetate as a result of photo AD. For that reason, a pH of 6, which is the most favourable pH for an anaerobic digester as indicated by the previous literature,^37^ was maintained throughout the operational incubation period using a 1 M NaHCO_3_ buffer solution. One of the main constraints causing low bioH_2_ yield or process stoppage in AD is maintaining low VFA concentration due to the high COD content of the influent. The VFAs, mainly acetate, accumulate inside the reactor and lead to unfavourable pH conditions that hinder maintaining a stable environment.^38^

**Fig. 5:**
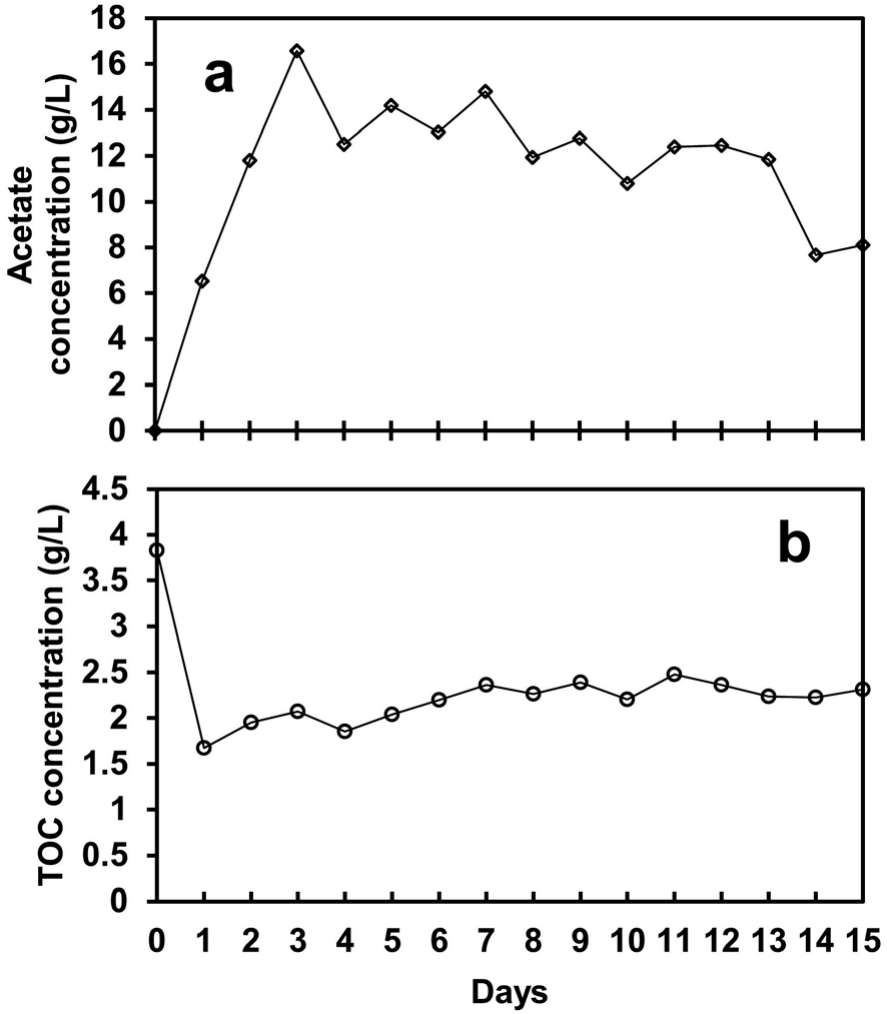
The acetate (VFA) (a) and total organic carbon (TOC) (b) profiles for the whole operation period of the SFBR.

Fig. 5a shows the acetate concentration, which was lowest at 6.5 g L^−1^ on day 1 during the exponential phase and increased to the maximum of 16.6 g L^−1^ on day 3 during the lag phase, followed by an average of 11.9 g L^−1^ in the steady-state phase. During the lag phase at peak acetate concentration, the biogas production dropped to almost 2.6 mL L^−1^ day^−1^, which indicates that the accumulation of acetate inside the reactor halted the bioH_2_ generation. Afterward, at an average of 11.9 g L^−1^ of acetate, the bioH_2_ concentration increased to the maximum of 56.4 μmol L^−1^ in the steady-state phase. Although acetate is one of the by-products of glucose anaerobic fermentation, it can inhibit the process if it accumulates in a high concentration.^39^ An excessive concentration of N_2_ (as shown in Table 2) in the biogas stream was also found to promote high VFA yield during the SFBR operation, leading to low bioH_2_ production.^21^

The TOC content also followed the same pattern as the acetate concentration mentioned above. During the exponential phase, the TOC concentration dropped to the lowest value of 1.7 g L^−1^ from 3.9 g L^−1^ on day 1, as shown in Fig. 5b. During the lag phase, when no bioH_2_ production was observed, the TOC content showed a slight increase in concentration of 20.7 mg L^−1^, as shown in Fig. 5b. This fact can be explained by the maximum acetate accumulation in the lag phase, partial inhibition of fermentation metabolism, and heterotrophic microalgae growth by acetate and organic carbon (glucose) uptake. Furthermore, as indicated by there still having enough organic carbon in the medium, the system did not produce bioH_2_, which suggests that organic carbon is likely to be consumed by the microbial community, specifically microalgae, for growth. The presence of microalgae in the effluent was observed daily throughout the incubation period, as evidenced by the green colour. Similarly, some of the evidence from previous studies verifies the presence of microalgal cells in the effluent of the digester tank after 30, 45, and 180 days of HRT.^40,41^

### 3.5. Energy conversion efficiency (ECE) of the H_2_ production process

To further evaluate the bioH_2_ production of the co-culture system using the SFBR, energy conversion analysis was performed to evaluate the conversion of glucose into bioH_2_ by anaerobic fermentation. The energy conversion efficiency (ECE) was calculated by evaluating the heat values generated by bioH_2_ and the amount of glucose being fermented by the bacteria according to the following equation:^20,42^
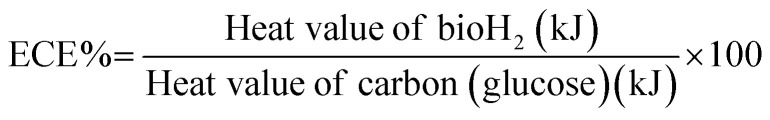


The heat values of H_2_ and glucose are calculated according to the following equations:^43^Heat value of bioH_2_ = *m*_H_2__ × *E*_H_2__Heat value of glucose = *m*_glucose_ × *E*_glucose_where *m* represents the mass of the bioH_2_ produced and the amount of glucose or carbon substrate added to the co-culture. And *E* represents the energy density for bioH_2_ and glucose taken as 142 kJ g^−1^ and 15.6 kJ g^−1^ as obtained from the previous studies.^44^

The energy conversion analysis presented in Table 3 presents the daily data for ECE. It is evident from the results that 12.9% ECE was achieved in the exponential phase during SFBR operation on day 1 which showed the highest conversion of glucose energy into bioH_2_ energy. The TOC concentration also reduced to the lowest value of 1.7 g L^−1^ on day 1, as shown in Fig. 5b, indicating that 2.3 g TOC L^−1^ was consumed for conversion into bioH_2_ out of 4 g TOC L^−1^ at the start of the experiment, while the rest of the bioH_2_ energy generated on day 1 might be due to the effect of microalgal metabolism. However, the ECE during the lag phase and steady-state phase remained negligible till day 7, whereas the ECE during the rest of the steady-state phase remained 0.1 and 0.2% which is also when the minimum amount of energy was converted into bioH_2_.

**Table 3:** Energy conversion efficiencies of bioH_2_ production using microalgae (*C. Vulgaris* CCALA 256) and activated sludge co-culture during SFBR operation

Days	BioH_2_ yield (mL L^−1^)	ECE (%)
0	0	0
1	169.5	12.9
2	0	0
3	0	0
4	0	0
5	0.1	0
6	0	0
7	0	0
8	0.9	0.1
9	1.2	0.1
10	2	0.2
11	1.2	0.1
12	2	0.2
13	2.1	0.2
14	1.3	0.1
15	1.2	0.1

In comparison to traditional fuels, a conversion efficiency of less than 10% is seen as less desirable.^42^ Despite the fact that the energy conversion efficiency observed in the present study is low, especially during the steady-state phase, the findings suggest that the biological H_2_ generation process can be made feasible by optimizing the process control parameters. Regarding ECE, it is difficult to compare this process to others reported in the literature since the outcome is very dependent on the substrate type and the composition of the inoculum (*C. vulgaris* and wastewater activated sludge).

### 3.6. Kinetic model fitting for SFBR biogas production

**Fig. 6:**
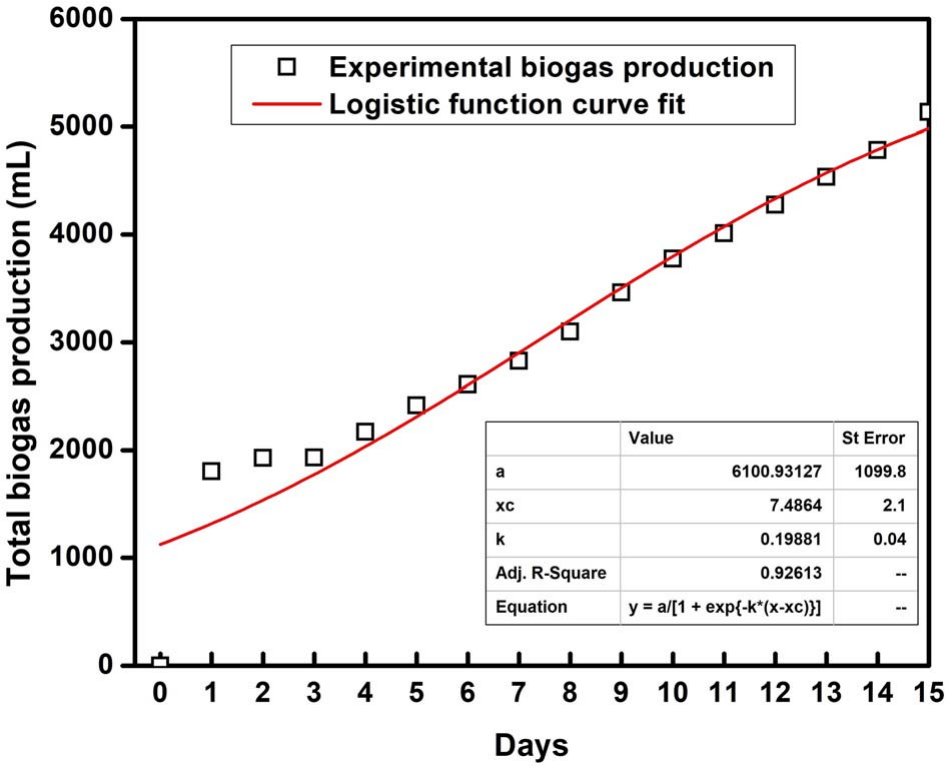
The logistic model fit for biogas production using the SFBR.

A logistic function model was used to evaluate the biogas production for the SFBR and showed the best fit during the incubation period of 15 days as shown in Fig. 6. Typically, the modified Gompertz fit and the logistic fit models are used to conduct the kinetic study of biogas production in batch and sequencing batch reactors.^45–47^ The logistic function fit used in the current study for the best fit prediction model of biogas production for the SFBR was modelled using the following equation as:
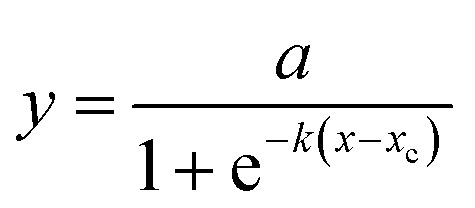
where *y*: the total biogas production (mL); *a*: the maximum biogas production potential (mL); *k*: the maximum biogas production rate or logistic growth rate (mL h^−1^); *x* and *x*_c_: the logistic domain (time) and sigmoidal midpoint.

The correlation coefficient obtained from the logistic model fit was 0.92 which suggests that the logistic model was the best fit for predicting the biogas production potential based on the experimental data for the SFBR.

## 4. Conclusions

The amount of sequential bioH_2_ produced by microalgae and WWAS co-culture using an SFBR was found to be inadequate yet consistent. The bioH_2_ concentration during the exponential phase was found to be sufficient at 421.1 μmol L^−1^; however, this concentration noticeably dropped down to 28–56.4 μmol L^−1^ for an HRT of five days and an OLR of 4.7 g COD L^−1^ day^−1^. Although the sequential bioH_2_ yield was consistent throughout the incubation period during the steady state phase, the HRT and OLR must be optimised for an efficient and improved bioH_2_ yield. Acetogenic metabolism abruptly raised the acetate concentration to the peak saturation level of 16.6 g L^−1^ in just one day, thus possibly overloading the system. Varying the OLR of considerable biomass may desiccate the medium and shift the microbial community, favouring enhanced bioH_2_ production. Furthermore, operating the SFBR and improving the conditions may make this reactor more practical than the constraints that make CSTR handling difficult.

## Author contributions

Muhammad Asad Javed: conceptualization, data curation, formal analysis, investigation, methodology, validation, visualization, writing – original draft; Ashraf Aly Hassan: conceptualization, funding acquisition, investigation, project administration, supervision, writing – review & editing.

## Conflicts of interest

There are no conflicts to declare.

## Supplementary Material

RA-012-D2RA06014K-s001
