# Supplementary material for: Photo fermentative biohydrogen production potential using microalgae–activated sludge co-digestion in a sequential flow batch reactor (SFBR)
Source: RSC Adv. 2022 Oct 18;12(46):29785–92. doi: 10.1039/d2ra06014k (PMC9577477; doi:10.1039/d2ra06014k)
Supplement: RA-012-D2RA06014K-s001 [file RA-012-D2RA06014K-s001.pdf]

Electronic Supplementary Material (ESI) for RSC Advances.
This journal is © The Royal Society of Chemistry 2022

Photo fermentative biohydrogen production potential using microalgae-activated sludge co-digestion in sequential flow batch reactor (SFBR)†

Muhammad Asad Javed [ab] and Ashraf Aly Hassan [1ab]

[a] Department of Civil and Environmental Engineering, United Arab Emirates University, Al Ain 15551, UAE; 201990206@uaeu.ac.ae, alyhassan@uaeu.ac.ae

[b] National Water and Energy Center, United Arab Emirates University, Al Ain 15551, UAE

[1] Department of Civil and Environmental Engineering, United Arab Emirates University, Al Ain 15551, UAE.
e-mail: alyhassan@uaeu.ac.ae

Figure S1

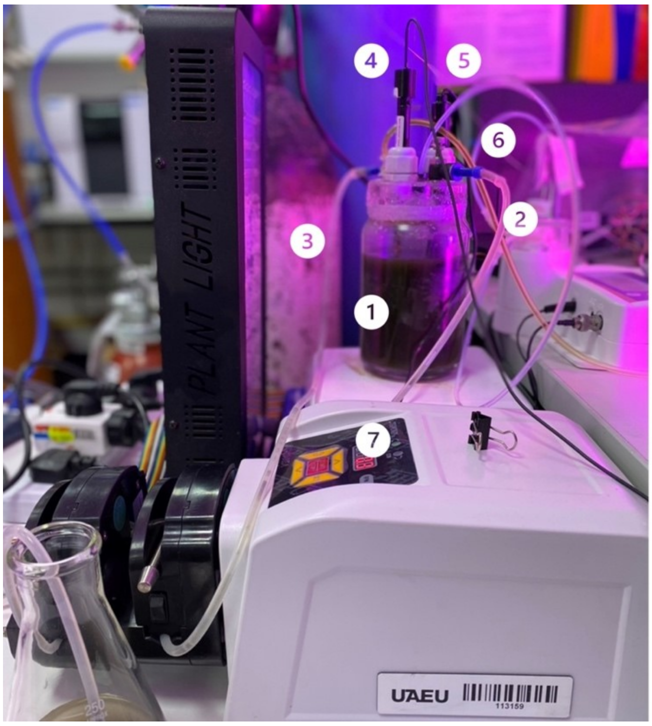

1. SFBR
2. Influent
3. Effluent
4. H_2 microsensor
5. pH controller
6. Gas port
7. Peristaltic pump

Figure S1.

Table S1. Nutrients for Z-medium

Component	Stock Solution (g/L dH_2O)[2]	Quantity Used (to 1 litre)
$NaNO_3$	46.7	10 ml
$Ca(NO_3)_2.4H_2O$	5.9	10 ml
K_2HPO_4	3.1	10 ml
$MgSO_4.7H_2O$	2.5	10 ml
Na_2CO_3	2.1	10 ml
Fe-EDTA solution	see following recipe	0.2 ml
Trace metals solution	see following recipe	0.08 ml

Fe-EDTA solution

Component	Quantity Used (to 250 mL dH_2O)
HCl (35%)	2.2 ml
$FeCl_3.6H_2O$	4.5 g
Na_2EDTA	4.65 g

Trace metals solution

Component	Quantity Used (to 250 mL dH_2O)
H_3BO_3	0.775 g
$MnSO_4.4H_2O$	0.4225 g
$Na_2WO_4.2H_2O$	0.022 g
$(NH_4)_6.MoO_{24}.4H_2O$	0.0297 g
KBr	0.044 g
$ZnSO_4.H_2O$	0.0365 g
$Co(NO_3)_2.6H_2O$	0.03125 g
$CuSO_4.5H_2O$	0.0592 g
$AlK(SO_4)_2.12H_2O$	0.775 g

[2] dH_2O as deionized or distilled water

Table S2. Nutrients for TAP medium

Component	Amount Used (to 2 litre)	Stock Solution Recipe	Amount
Beijerinck's Solution	100 mL		
		NH_4Cl	8 g/L
		$CaCl_2.2H_2O$	1 g/L
		$MgSO_4.7H_2O$	2 g/L
Phosphate Buffer Stock Solution	17 mL		
		Na_2HPO_4	11.62 g/L
		KH_2PO_4	7.26 g/L
Hunter's Trace Stock Solution	2 mL		
		$Na_2EDTA.2H_2O$	50 g/L
		$ZnSO_4.7H_2O$	22 g/L
		H_3BO_3	11.4 g/L
		$MnCl_2.4H_2O$	5.1 g/L
		$FeSO_4.7H_2O$	5 g/L
		$CoCl_2.6H_2O$	1.6 g/L
		$CuSO_4.5H_2O$	1.16 g/L
		$(NH_4)_6Mo_7O_{24}.4H_2O$	1.1 g/L
Tris Acetate Stock Solution	20 mL		
		Trisma Base	121 g/500 mL
		Glacial Acetic Acid	50 mL/500 mL
